# Need for speed: Short lifespan selects for increased learning ability

**DOI:** 10.1038/s41598-019-51652-5

**Published:** 2019-10-23

**Authors:** Jannis Liedtke, Lutz Fromhage

**Affiliations:** Department of Biological and Environmental Science, University of Jyvaskyla, PO Box 35, Jyvaskyla, 40014 Finland

**Keywords:** Behavioural ecology, Evolution, Animal behaviour

## Abstract

It is generally assumed that an investment into cognitive abilities and their associated cost is particularly beneficial for long-lived species, as a prolonged lifespan allows to recoup the initial investment. However, ephemeral organisms possess astonishing cognitive abilities too. Invertebrates, for example, are capable of simple associative learning, reversal learning, and planning. How can this discrepancy between theory and evidence be explained? Using a simulation, we show that short lives can actually select for an increase in learning abilities. The rationale behind this is that when learning is needed to exploit otherwise inaccessible resources, one needs to learn fast in order to utilize the resources when constrained by short lifespans. And thus, increased cognitive abilities may evolve, not despite short lifespan, but because of it.

## Introduction

Despite enormous scientific effort, the evolution of increased cognitive abilities is still puzzling in some respects. Benefits of e.g. associative learning e.g.^1^, social competence^2^, or tool-use e.g.^3,4^ are easy to comprehend and numerous examples of such abilities are found in diverse taxa. However, these behaviors are associated with an investment in neuronal tissue underlying these cognitive abilities. Neuronal systems are metabolically extremely expensive^5,6^ therefore raising the question what ecological conditions make this investment worthwhile. This gave rise to the idea that an investment in cognitive abilities is particularly beneficial for long-lived species, as a long lifespan offers more opportunities to recoup the initial investment (sometimes referred to as “delayed benefits hypothesis”^7–9^). Indeed, many studies superficially support this explanation, showing a positive correlation of enlarged brains, cognitive abilities and longevity e.g.^8,10–13^. Some studies have speculated that a prolonged life is primarily caused by a slowed development due to the slow growth of the brain^12,14^ and an increased period in which individuals have to practice the skill for which the cognitive abilities have evolved (reviewed in e.g.^8,15,16^). Nonetheless, the “delayed benefits hypothesis” is considered to describe a major factor in the evolution of animal intelligence, that may explain the correlation between enlarged brains and longevity e.g.^7,9^.

However, also relatively “simple” and ephemeral organisms possess cognitive abilities formerly thought to be exclusive to long-living and large-brained vertebrates. Nematodes, for example, are capable of simple associative learning (reviewed in e.g.^17^). Arthropods such as insects or spiders show learning and reversal learning abilities e.g.^18–20^, planning^21^ and social learning^22^. Similar, octopus species with lifespans mostly not larger than 2 years show extraordinary skills in part comparable to those found in primates, corvids and other “brainy” vertebrates (reviewed in^23–25^).

How can this apparent mismatch between theory and evidence be explained? We propose that a previously overlooked selection pressure imposed by longevity on learning speed may play a role. Using an individual-based simulation and an analytical model we show that short lives can actually select for an increase in cognitive abilities in terms of learning speed. And thus, increased cognitive abilities may evolve, not despite short lifespan, but because of it. The rationale behind this is that, when learning is needed to exploit otherwise inaccessible resources, one needs to learn fast in order to utilize the resources when constrained by short lifespans.

Evidently an investment in neuronal machinery i.e. the brain is costly^26,27^ and should only be made if needed. Some experimental studies were able to show a change in brain size or brain compartments with a change in environmental complexity^28–30^ and other studies could show a rapid response of brain size to artificial selection regimes^31^. These findings suggest that in nature, too, brain size may readily evolve in such a way as to suit a species’ present ecological conditions. For example, since a long life may provide sufficient opportunities even for slow learners to master cognitive challenges, long-living species may be able to reduce their investment in learning speed. Thereby, environmental conditions affecting longevity may indirectly affect the need for fast learning and the corresponding investment in neuronal tissue.

To test this idea, we use an individual based simulation in which learning speed evolves according to its effect on individuals’ lifetime success in gathering resources, in an environment where resources differ in how much learning is required to be able use them. We run simulations with different season length to investigate the influence of longevity on investment into learning speed. In addition, we present an analytical model that illustrates the same key principle in a simplified form (see Supplementary Methods “Analytical Model”).

## Material and Methods

### Individual based simulation

We model a population of size *N*_*individuals*_, in which learning speed *L* evolves for *N*_*generations*_ discrete generations (seasons). For an overview of our notation and default settings, see Table 1. At the end of each season, individuals reproduce asexually in relation to the amount of resources they have collected. Each season has *T* days, which determines the individuals’ lifespan. Each day, individuals are assigned in random order to proceed through *N*_*Steps*_ time steps.

**Table 1:** Notation and default settings.

Individual based simulation		Default setting
*L*	Learning ability = Learning speed	0–1
*T*	Season length = Lifespan	1–500
*h*_*i*_	Time to handle resource type i	t_1_, t_5_, t_17_
*R*_*i*_	Names for resource types i	R_1_, R_2_, R_3_
*V*_*i*_	value of resource i	V_1_, V_5_, V_15_
*P*_*i*_	Probability of find resource type i in a patch	5/22
*N*_*Days*_	Number of days per season	1–340
*N*_*Steps*_	Number of time steps per day	5
*N*	Population size	200
*G*	Number of generations	200
*α*	Cost coefficient that specifies the cost of learning	1/1.4

The default settings were chosen after explorative analysis, in order to provide a clear picture of the mechanism we wish to illustrate.

#### Sites and resources

We model the environment as being composed of *N*_*sites*_ ‘sites’, i.e. locations where individuals encounter resources. In the beginning of each day, each site is randomly assigned (with probability *P*_i_) to contain a single resource item of type *R*_i_. Resource types are characterized by their handling time *h*_i_ and their resource value *V*_i_. Empty sites are modelled as containing resource type *R*_0_, with handling time *h*_0_ = 0 and value *V*_0_ = 0.

#### Actions

There are two types of actions which an individual can perform during a time step: visit a site, or handle a resource. In the first time step of each day, each individual visits a randomly selected (unoccupied) site. In the following time step, an individual may either move to another site or stay in the present site to handle the encountered resource item. If the individual handles a resource item, it reduces the item’s residual handling time by one unit per time step. When an item’s residual handling time is reduced to zero, the individual collects the item (i.e. it adds the corresponding value *V*_i_ to its total) and moves to a new site in the following time step. When the day ends before the residual handling time has been reduced to zero, the item is not collected.

#### Learning

We implement learning as a reduction of handling time due to having experience with handling a given type of resource. Specifically, every time an individual ends handling a resource item of type *R*_i_, we update the handling time for this individual and this type of resource type as ${h}_{{\rm{i}}}=\,{\rm{\max }}\,[1,\,{h}_{{\rm{i}}}-t\cdot L/{h}_{{\rm{i}},{\rm{initial}}}]$. Here, *L* is the focal individual’s learning speed; *t* is the number of time steps spent handing the resource item; *h*_i,initial_ is the initial handling time for resource type *R*_i_ at the beginning of the current encounter; and the maximization function max[.] ensures that handling times cannot drop below 1. We let *L* be genetically encoded by a single locus, whose initial allelic values are randomly sampled from a uniform distribution between 0 and 1.

#### Selectiveness

We model individuals as being either *selective* or *non-selective* foragers.

*Selective* individuals handle only resources whose handling time they can complete by the end of the day. When they encounter other resources, they immediately move to a new site in the next time step. *Non-selective* individuals handle all resources they encounter. This can lead to handling times being interrupted prematurely at the end of the day, without yielding any immediate reward. However, such interrupted handling times still provide an opportunity for learning. As a result, *non-selective* individuals can eventually learn to collect resource types whose initial handling times did not fit into a day. We let selectiveness be genetically encoded by one locus with two alleles, which determine that individuals are either *selective* (*S* = 1) or *non-selective* (*S* = 0). The initial allelic values are randomly sampled with equal probability.

#### Reproduction

At the end of each season, we calculate each individual’s reproductive success as *F* = *V*_total_ * (1-*α* * *L*), where *V*_total_ is the total value of its collected resources, *L* is its learning speed, and *α* is a cost coefficient that specifies the cost of learning. In other words, the reproductive success is reduced in proportion to the investment in learning speed. This implementation is based on the assumption that learning is costly and any investment in this trait will therefore be traded-off with other investments which eventually translate into reduced fecundity. The assumption of a proportional cost reflects the idea that increasing amounts of neuronal tissue (corresponding to increasing learning speed) may account for increasing proportions of an animal’s metabolic expenditure.

The next generation is recruited by randomly sampling *N*_*individuals*_ new individuals from the present generation’s offspring, using *F* as the (independent) sampling probabilities.

#### Mutation

Both traits, *L* and *S*, were independently subject to mutation. Mutation probability was set to *q* = 0.1 for each trait. For the continuous trait *L*, new trait values were chosen randomly from a Gaussian distribution with a mean of the parental trait value and a SD of 0.1.

## Results

**Figure 1:**
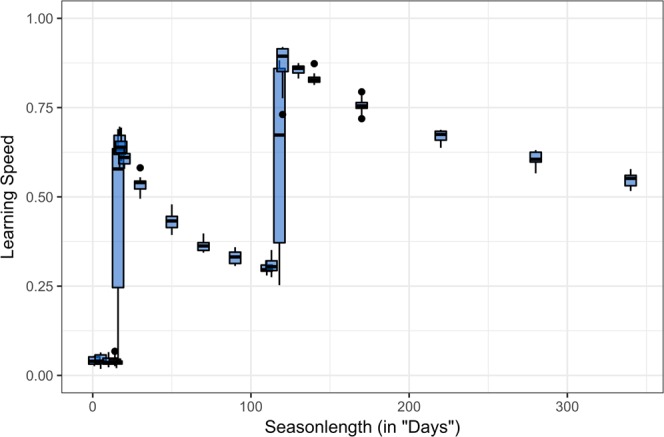
Relationship between investment in learning speed and lifespan: Simulation model. Each box-plot is based on 10 simulations with 200 individuals and 200 generations. Whiskers cover values no further than 1.5 * IQR from the hinges. The simulated environment contained three different resource types (R_1_, R_2_, R_3_) which could be collected by individuals. R_1_ could be processed without learning. R_2_ and R_3_ could only be processed after a certain learning period which was small for R_2_ and larger for R_3_. The first peak in investment into learning is a consequence of that the corresponding lifespan was long enough to master the cognitive challenge of R_2_ and to recoup the investment into learning. The second peak was the point at which individuals with high investment in *L* could solve R_3_. The decline in investment in *L* after each peak is caused by the fact that the total amount of collected resources increases with lifespan and thus in relation the benefits of obtaining x resources more diminished. Under these circumstances investing into high learning speeds becomes less attractive with increased lifespans and slowly reaches a minimum as seen by the asymptotic decline of mean *L*.

In the simulations we implemented three different resources. One resource could be obtained without learning (*R*_1_) which had the lowest value. Resource *R*_2_ and *R*_3_ had higher values and correspondingly higher handling times, and hence could be obtained only after a certain amount of reduction in their handling-time through learning. In order to successfully collect these resource types, individuals needed to invest into *L*. An investment into *L* started to be beneficial with a minimal season length which allowed to successfully process resource type *R*_2_ (first peak in Fig. 1). With a further increase in season length, investment into learning speed was reduced again, since slower learning in a longer life still allowed to learn to handle this resource and the reduced cost of learning outweighed the benefits of faster learning.

We can observe a second peak in investment into learning speed with increasing season length. At this point, a high learning speed allowed to successfully process resource type *R*_3_. Again, a further increase in season length causes a decline in *L* towards some minimum.

The large difference between first and third quartiles (i.e. the 25th and 75th percentiles) of the box-plots at a season length of 16 and 118 is caused by some simulation runs converging to either high or low values of *L*. In some runs the final population in generation 200 consists of two behavioral types (“cognitive styles” sensu e.g.^32^): selective individuals specializing on exploiting resource *R*_1_ and investing little into learning and non-selective individuals strongly investing into learning speed and being able to exploit resource *R*_2_ (at *T* = 16) or *R*_2_ and *R*_3_ (at *T* = 118; see Supplementary Figs S2–S5 for detailed visualization). The coexistence of different cognitive styles is noteworthy in view of the recent interest into the evolution of individual differences in cognitive performance within populations see e.g.^33^. Increasing or decreasing the cost of learning did not change the general pattern of investment into *L*, but higher cost lead to a later onset and a generally lower magnitude of investment into learning speed (see Supplementary Fig. S6).

## Discussion

**Figure 2:**
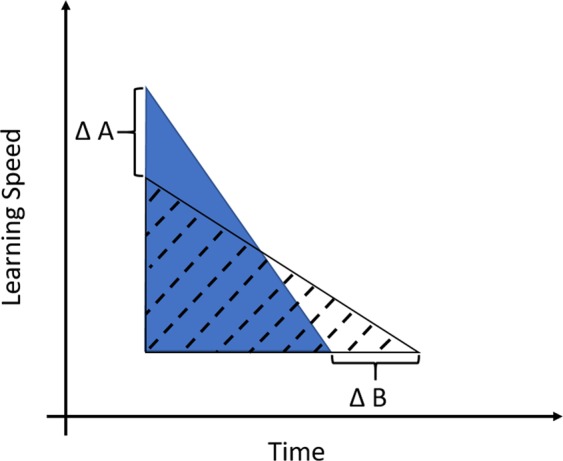
Schematic representation of alternative (fast vs. slow) learning strategies. The areas of triangles represent the learning effort needed to solve a cognitive challenge. Each triangle represents a different strategy: one fast learning individual or species (Type A, dark blue) and one slower learning (Type B, dashed). The size of the triangles’ areas are identical, to indicate that the same learning effort (hence the resulting skill level) is equal for both strategies. The bottom right corner of each triangle represents the point in time (x-axis) when an individual masters a cognitive challenge. Investment into cognitive abilities (here learning speed) is represented on the y-axis. The upper left corner represents the investment in learning speed. Delta A is the difference in investment into learning speed between Type A and Type B. Delta B is the time difference until mastering the cognitive challenge. We expect that individuals or species increase learning speed or time until mastering the cognitive challenge depending on the relation between the cost and benefit of each trait. For example, a given absolute investment into neuronal tissue might be relatively cheaper for larger than for smaller species due to metabolic costs. On the other hand, in species with parental care it might be “cheaper” to increase the time to master a challenge (and thus increase ∆B). However, lifespan will place an upper limit for increasing the learning/development period as individuals will need some minimum time to recoup the investment once mastering the challenge. Thus, short-lived species might be under particular strong pressure to invest into learning speed.

Based on our simulations, and supported by the analytical model (see Supplementary Methods “Analytical Model”), we found that shorter lifespans can select for increased investment into learning speed. We want to emphasize that this finding does not necessarily contradict the logic that a long lifespan can help to recoup investment as postulated e.g. by the “delayed benefit hypothesis”^7–9^. However, we suggest that the effect of longevity on cognitive abilities and enlarged brains can work in opposing directions. On the one hand, by increasing the time period for recouping the investment, longer lives may indeed facilitate the evolution of cognitive abilities. On the other hand, by limiting the time available for learning, short lives impose an incentive to either learn particularly fast or not at all. Which mechanism predominates depends on ecological details, as we discuss below (see Fig. 2).

In general, we still expect a positive correlation of learning speed and longevity when increased lifespans are associated with *additional* cognitive challenges faced by individuals which leads to *additional* investment into cognitive abilities. This expectation is in line with studies pointing out that a prolonged life will increase the likelihood to encounter some kind of “crisis”^11^, or of having to deal with a changing environment^7^, compare^8^. However, once all cognitive challenges have been mastered in a stable environment, a further increase in lifespan may relax the selection on learning speed since being a little faster will likely not outweigh the investment into enhanced learning.

These results may help to explain why some short-living species seems to be extraordinarily intelligent i.e. showing higher cognitive abilities than expected based on their life-history. If species live in complex environments in which certain cognitive abilities are beneficial or even necessary, we expect these species to evolve those abilities or to be driven out of this niche; compare e.g. the suggested evolutionary pathways in cephalopods reviewed in^23–25^. One possibility to realize this is through stronger investment into learning, as shown by the models described here. An alternative evolutionary pathway (not modelled here) towards coping with cognitive challenges could be to increase lifespans. The latter may allow solving cognitive challenges with an extended learning period and a long enough “post-learning” period in which the investment can be recouped (“delayed benefits hypothesis”). While such an extended period of learning is likely to be costly in itself, some mechanism such as parental care has been proposed to “buffer” these costs and allow for the development of more sophisticated skills which need a long time to be learned and to reach adult performance levels e.g.^14,15^. Yet, whether parental care is a consequence or driver for the evolution of increased cognitive abilities is a matter of debate see e.g.^9^. In general, we may expect short-lived animals to solve some cognitive tasks faster than slightly longer-lived species from a similar niche. This is because short-lived species may be under stronger selection for fast learning. In order to limit confounding variables, it would be helpful to conduct such comparisons between closely related taxa, e.g. by investigating if some parrotlets are faster in associative learning than macaws.

We caution that these considerations do not apply in cases in which cognitive abilities are needed for direct interactions with conspecifics e.g. deception and cooperation^34^ and Ref. therein, or functioning as a fitness signal (e.g. bird songs^35^), or in competition with other species (e.g. predator-prey-interactions^36^). In these cases an evolutionary arms race can be expected, leading to high investment into cognitive abilities even with prolonged lifetime.

However, we want to stress that longevity per se does not select for increased learning abilities but instead may relax the selection for learning. Furthermore, in line with the mechanism explained in the present study, the logic that a long life enables individuals to recoup initial investment into cognition (i.e. “delayed benefits hypothesis”) is incomplete and needs to be formulated more precisely. A long life can, on the one hand, allow individuals to recoup their initial investment. But at the same time, it also relaxes the selection pressure for learning speed, because a learning phase of given absolute duration (during which the individual is not yet a fully efficient forager) becomes proportionally shorter in relation to an increased lifespan. This relative reduction of learning period in longer lives translates to a reduction of the cost of the learning period. In other words, in a long life it matters less if one needs some days more to master a cognitive challenge. Thus, whether a prolonged life will select for higher investment into learning, as it facilitates recouping the investment, or whether it decreases the investment into learning speed, as longer learning periods may matter less, will crucially depend on how the ecological context shapes the costs and benefits of different learning strategies (see Fig. 2). The higher the cost of investment into neuronal tissue, the more likely a prolonged life will shift the selection pressure towards a longer learning period. The more costly the learning period becomes, e.g. due to increased predation or fewer learning opportunities, the more likely the balance will be shifted towards increased learning speed.

The negative effect of lifespan on learning speed may be confounded by other effects such as that increased cognitive abilities may allow to survive longer (e.g. cognitive buffer hypothesis^7^). So, in the end it may be difficult to actually show the expected effects of our model since the evolution of learning abilities is not a univariate process but is influenced by multiple factors which may work in the same but also in opposing directions.

Finally, animals may face different cognitive challenges, which vary extremely in their complexity, i.e. from simple association learning to tool-use and theory of mind. We do not suggest any explanation for when to expect which quality of cognitive abilities are selected for, as this will crucially depend on the species’ ecology. However, we suggest that for each cognitive ability a similar relationship between investment and longevity might occur. Investment into learning speed for specific abilities is expected to be highest when the lifespan is just long enough to master the challenge, to recoup the initial investment and to gain a net benefit out of it. Any further increase of lifespan will relax selection and thus decrease the investment into this ability towards some minimum. This minimum will depend, among other things, on maximal lifespan, cost of cognition and available alternative resources or strategies that are less cognitively demanding.

## Conclusion

By highlighting the two-sided nature of the link between lifespan and investment in learning, we argue that the positive correlation between lifespan and enlarged brains in mammals and birds is just one of several possible evolutionary outcomes. The mechanism shown in this study should help to appreciate that a combination of fast life-history strategies and well-developed cognition (as increasingly documented species such as insects, spiders and cephalopods) should not be surprising but indeed can be expected. Understanding the selection pressures faced by short-living species, and the proximate solutions, such as the development of miniature brains with high computation powers, will not only shed light on the evolution of intelligence in these species but on animal intelligence in general.

## Supplementary information

Supplementary Material

## Data Availability

Code of simulation and data are available in dyrad (10.5061/dryad.k0p2ngf43.).
