# Supplementary material for: Need for speed: Short lifespan selects for increased learning ability
Source: Sci Rep. 2019 Oct 23;9:15197. doi: 10.1038/s41598-019-51652-5 (PMC6811680; doi:10.1038/s41598-019-51652-5)
Supplement: Supplementary file 1 — Supplementary Material [file 41598_2019_51652_MOESM1_ESM.pdf]

Supplementary Material to

Title: Need for speed: Short lifespan selects for increased learning ability.

Authors:

Jannis Liedtke[1*] and Lutz Fromhage[1]

Affiliations:

[1] Department of Biological and Environmental Science, University of Jyvaskyla, PO Box 35, 40014, Finland

*Correspondence to: jannisliedtke@gmx.de

Supplementary Methods "Analytical Model"

Here we consider a simplified version of our model in which there is a single time step per day (such that season length *T* equals the total number of time steps), and in which the same resource type (with value *V* and initial handling time h_{initial}) is encountered in every time step. If $h_{\text{initial}} > 1$, an individual can only begin to collect resource items after having reduced the handling time to $h = 1$ through learning. Specifically, *h* is reduced by *L* units per time step. This implies that, in the beginning of a season, there is a delay of duration $(h_{\text{initial}} - 1)/L$ before the collection of resource items begins. This delay limits the portion of the season available for resource collection to duration $\tau = T - (h_{\text{initial}} - 1)/L$. Since resources are collected at rate *V* per time step during this latter part of the season, the total amount of collected resources, expressed as a function of *L*, is $V_{\text{total}}[L] = V\tau$. We let the cost of learning (expressed as a function of *L*) be $C[L] = \alpha L$. Based on the above, we can express an individual's reproductive success as

$$F[L] = (1 - C[L])V_{\text{total}}[L] \qquad (1).$$

To find the optimal learning speed L^* that maximizes $F[L]$, we differentiate $F[L]$ with respect to *L*, set the derivative equal to zero, and solve for *L*. This yields:

$$L^* = \sqrt{h_{\text{initial}} - 1}/\sqrt{T} \qquad (2)$$

Plotted as a function of lifespan (*T*), this equation predicts a decrease of optimal (investment in) learning speed with increased lifespan (Supplementary Fig. S1 online). The biological rationale behind this result is that a slow learner's initial delay in collecting resources becomes increasingly irrelevant in the context of a long lifespan. Depending on the initial handling time h_{initial} (i.e., depending on the complexity of the cognitive task), L^* decreases more or less steeply towards an asymptote of zero.

Figure S1

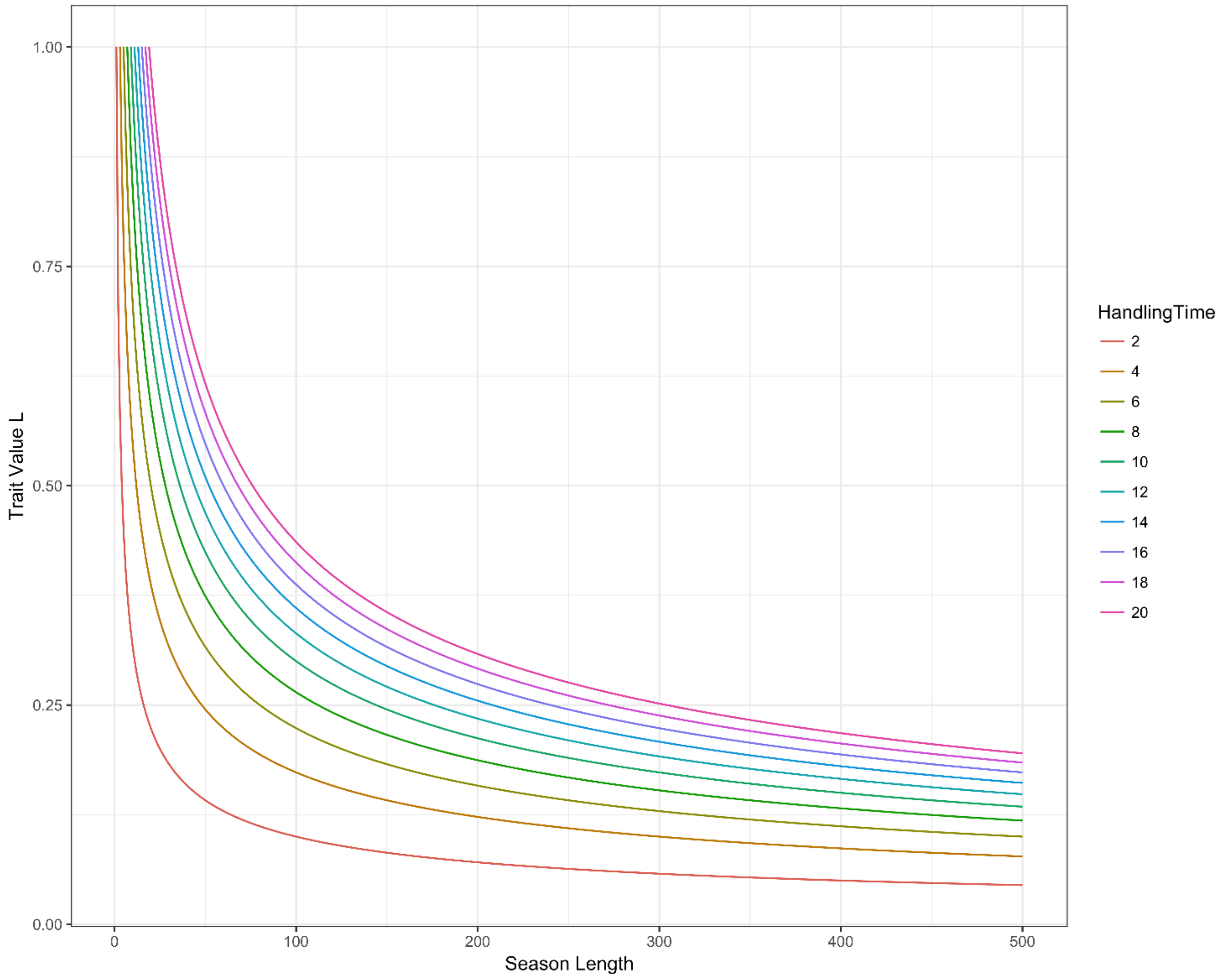

Fig. S1: Relationship between optimal learning speed and lifespan: analytic model

Optimal learning speed *L** declines with season length (lifespan). The shorter the handling time *h*, (i.e. the easier it is to learn the cognitive challenge), the faster *L** declines. The biological rationale behind this relationship is that, even with maximum investment into learning abilities, some minimum time is required in order to master a cognitive task and to recoup the investment (compare the 'delayed benefits hypothesis'). This minimum time is given by the season length at which *L**= 1. Any increase in season length beyond this minimum leads to a decline in investment because the cost of any given investment increasingly outweighs the benefits of obtaining additional resources early in the season. Since the total amount of gained resources increases with lifespan, the benefit of obtaining x additional resource items becomes increasingly negligible in relation to the total.

Figure S2

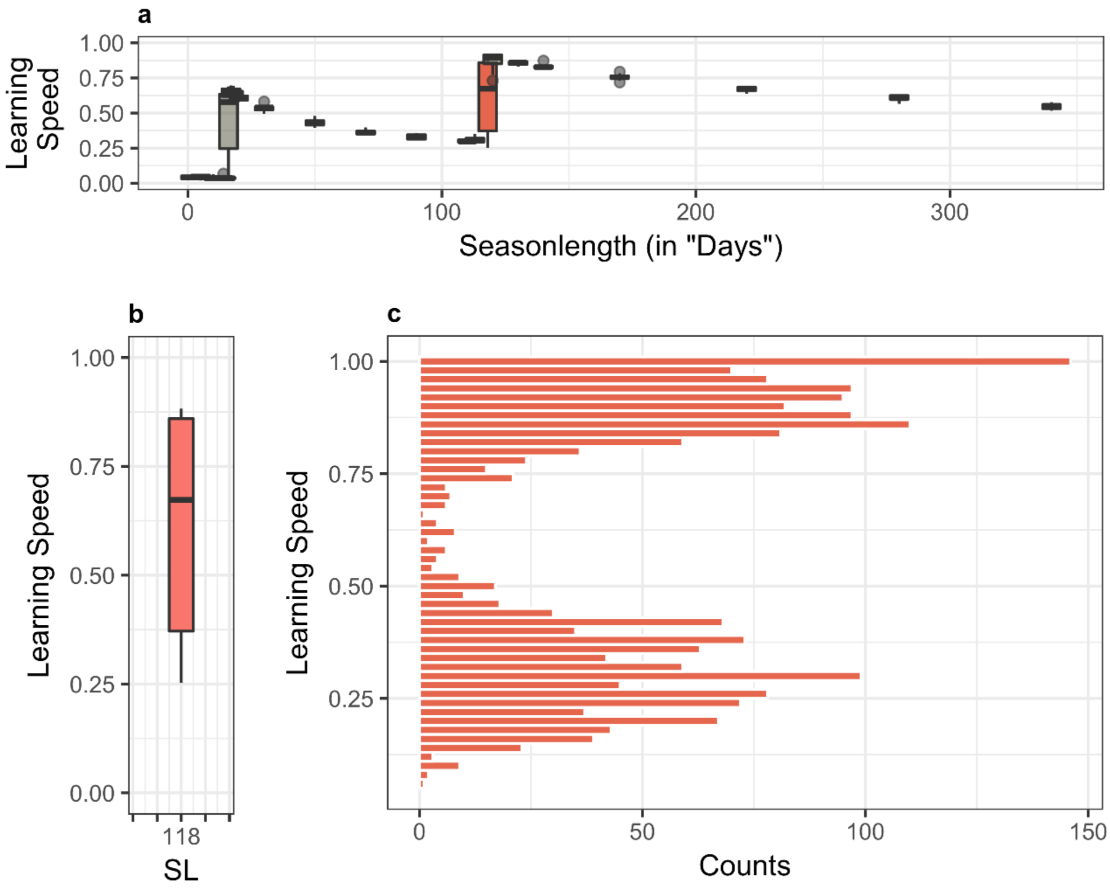

Fig.S2: Highlighting season length 118

A) is a replicate from Fig. 1 with the boxplot highlighted for a season length of 118 days. B) this boxplot showing its large variance. C) gives the count of individual values of `*L*´ pooled from all 10 replicated simulations. The two peaks represent two different strategies with one subset of individuals with moderate values of `*L*´ which specialized on obtaining resource type R_1 and type R_2 and another subset of individuals investing highly into learning speed which allows for processing resource type R_3 as well. Apparently, for a season length of 118 days, both strategies seem to lead to similar fitness outcomes (see Fig. S5) producing simulations ending with either high learning, low learning or also with a mix of both strategies.

Figure S3

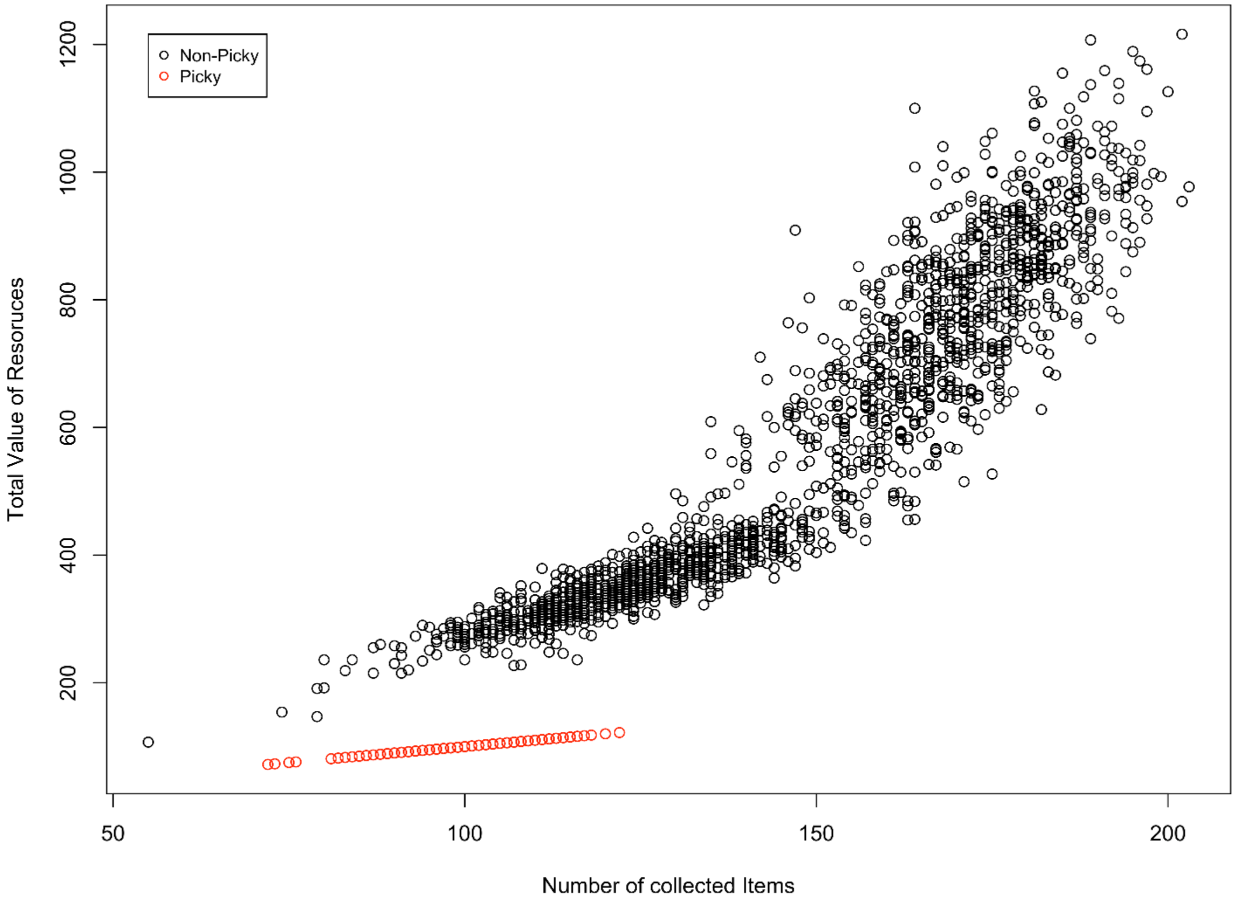

Figure S3: Individual number of collected resources and their total value

The circles represent the number and their total value of collected resource items for one individual each (red for picky and black for non-picky individuals) in a simulation with season length of 118 days. The linear increase (i.e. slope) is lowest for picky individuals, since they only obtain resources of type R_1 which had a value of 1. For non-picky individuals we can distinguish two different groups. One group of individuals were able to obtain resources types R_1 and through learning also R_2 with a value of 5. Consequently, we observe a steeper linear increase in the total value with increasing number of collected items than for picky individuals (lower left part of the black cloud). Individuals which could also process resources of type R_3 with values of 15 had the highest slopes (upper right part of the black cloud).

Figure S4

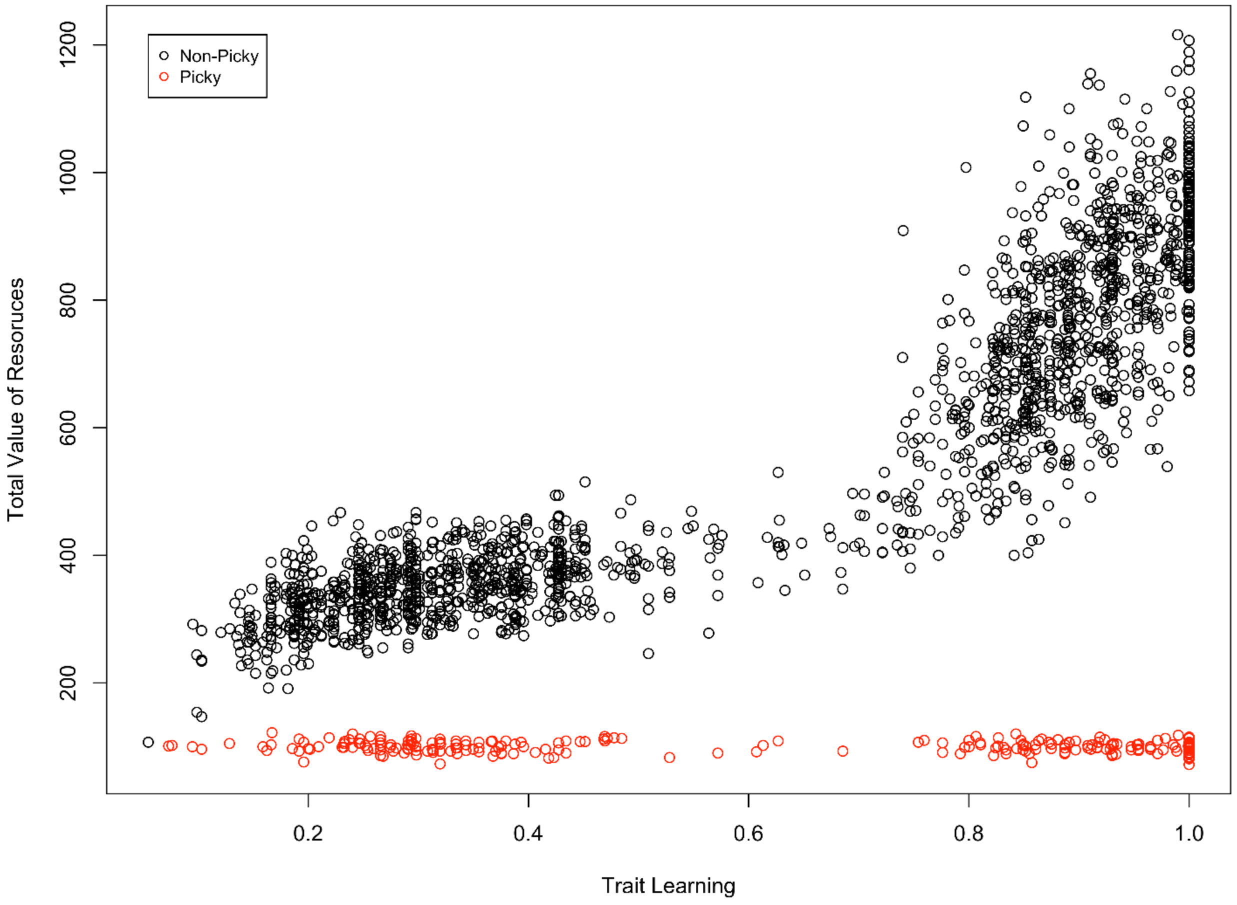

Figure S4: Total value of resources

The figure shows the total value of resources (y-axis) plotted against learning speed `*L*´ (x-axis) in a simulation with season length of 118 days. Picky individuals (red circles) accumulated the lowest value since they would never learn through experience (even with high `*L*´) and could process resources of type R_1 only. Non-picky individuals (black circles) with `*L*´ lower than roughly 0.6 were able to learn to process resources of type $R_2$ but not $R_3$. This led to an intermediate amount of obtained resource values. However, because these individuals had lower cost of `*L*` they could compete with individuals which invest high in `*L*´ and were therefore able to process R3 and gained the highest total resource values (see Fig. S5).

Figure S5

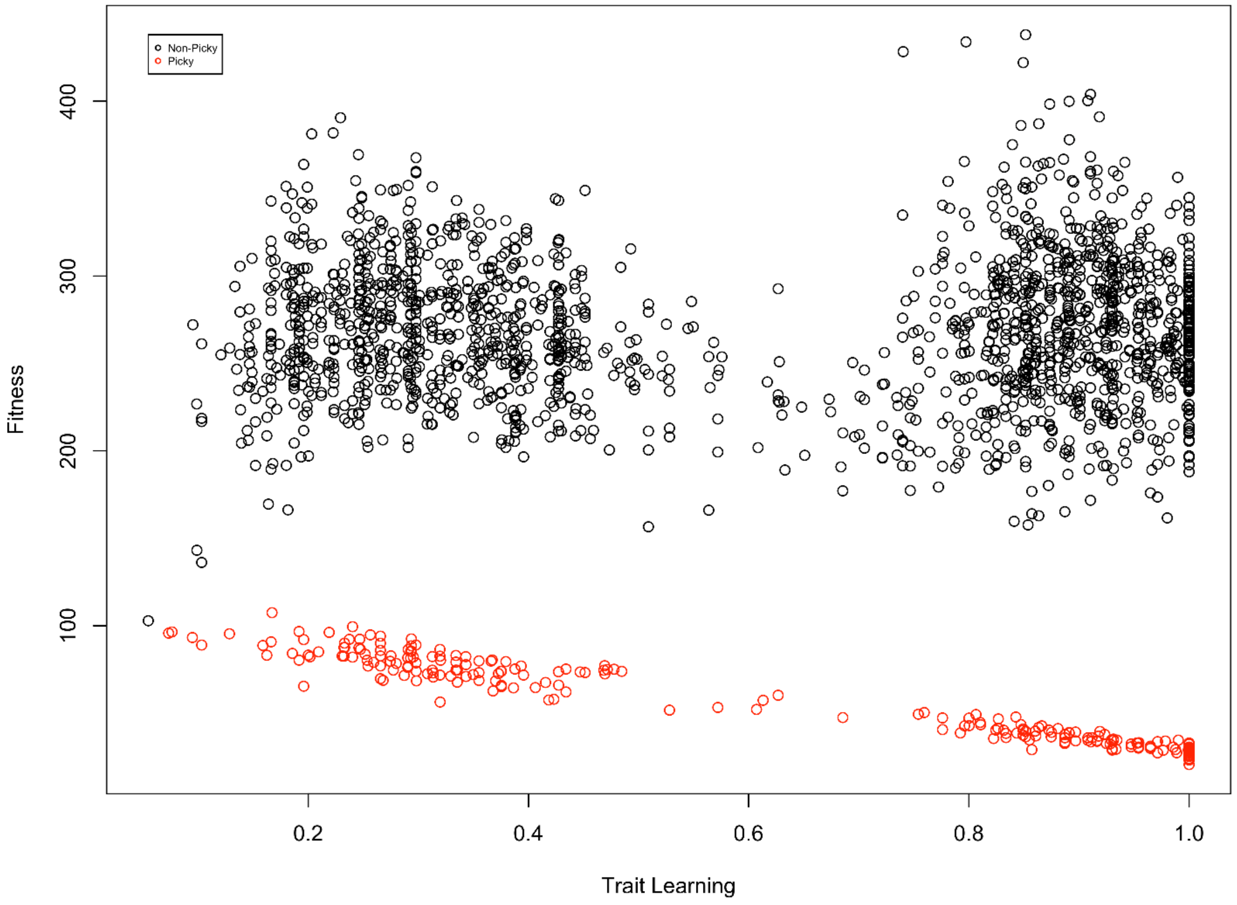

Figure S5: Fitness plotted against `*L*´

The figure shows the fitness of individuals plotted against `*L*´ in a simulation with season length of 118 days. Fitness was calculated by the accumulated value of processed resource items minus the cost for `*L*´ ($\sum$ value of collected resources * (1-α*L)). With the given season length, two strategies gave roughly the same fitness outcomes: one set of individuals invested strongly into `*L*´ and could process the highest valuable resource of type R3 but also had to pay the highest cost for their increased learning abilities. Another set of individuals moderately invested into `*L*´ which allowed them to process resource type R2 but not R3. Due to the lower cost of their moderate learning abilities they reached the same fitness as “smart” individuals. Picky individuals (red circles), never learned to solve R2 nor R3 and showed a negative correlation of fitness with increasing `*L*´. They only paid the cost for increased learning but never used it and therefore never gained the benefits of it.

Figure S6

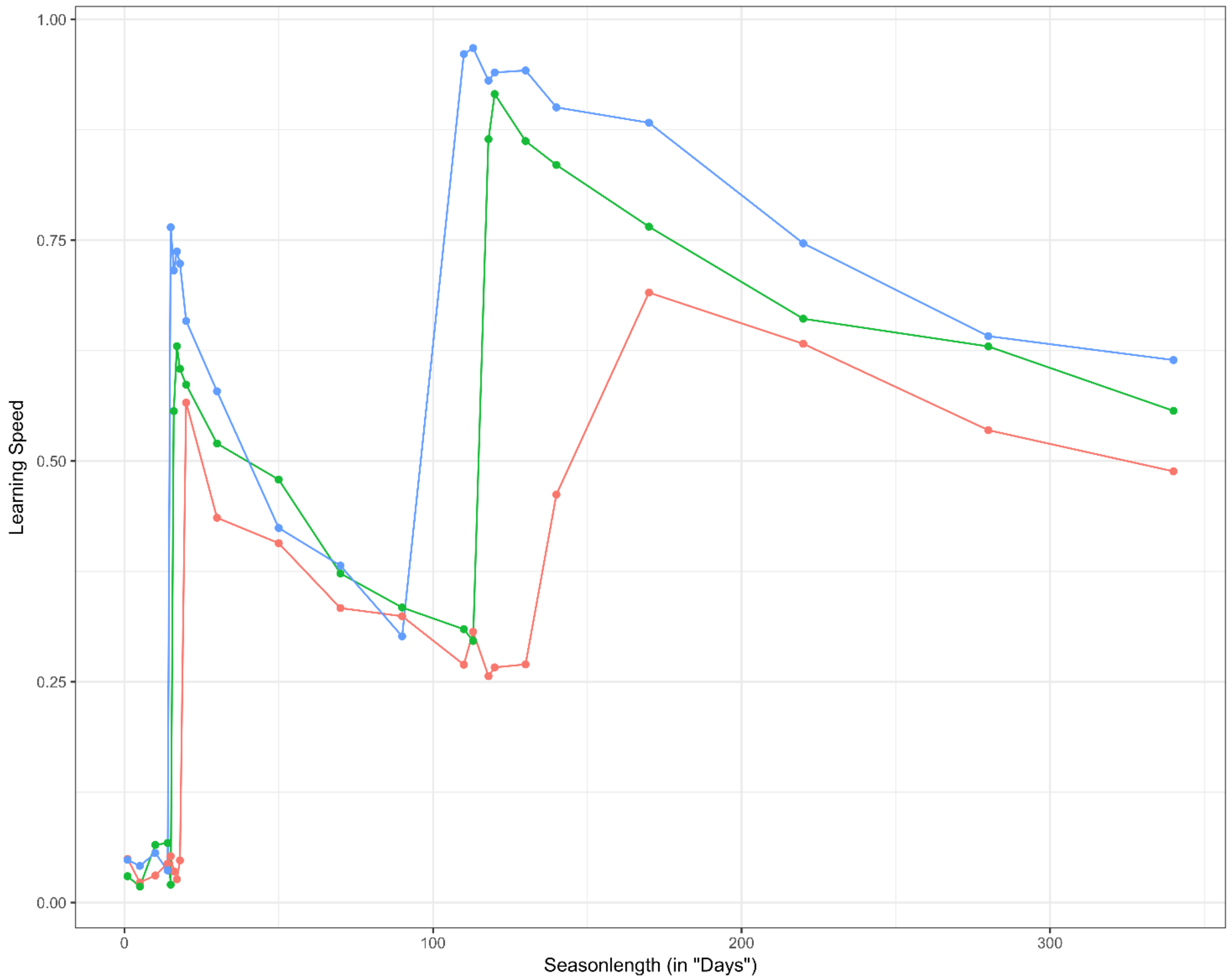

Figure S6: Different cost for `*L*´

The figure shows the result of three sets of simulation with different cost (different values for *alpha*) for learning speed (`*L*´). The blue line shows the relationship between investment into `*L*´ and season length for relatively low cost (∑ value of collected resources * (1-α*L)). For the green and the red line, the cost for `*L*´ were higher. The increase in cost of `*L*´ results in two changes: 1) the maximum investment into `*L*´ is reduced and 2) and the investment into `*L*´ begins only with longer season length. The later onset of investment into `*L*´ is likely to counterbalance the increased cost of `*L*´ for which one needs to obtain more high valued resources. This only can be done with increasing lifespan (as predicted by the “delayed benefit hypothesis”).
